# A Novel Multiobjective Control of DVR to Enhance Power Quality of Sensitive Load

**DOI:** 10.1155/2015/385109

**Published:** 2015-11-03

**Authors:** Sathish Babu Pandu, Kamaraj Nagappan

**Affiliations:** ^1^Department of Electrical and Electronics Engineering, University College of Engineering, Panruti, Tamil Nadu, India; ^2^Department of Electrical and Electronics Engineering, Thiagarajar College of Engineering, Madurai, Tamil Nadu, India

## Abstract

The Dynamic Voltage Restorer (DVR) is one of the fast, flexible, and cost effective solutions available in compensating the voltage-related power quality problems in power distribution systems. In this paper is discussed how power quality enhancement of sensitive load is achieved by applying three versions of Autonomous Group Particle Swarm Optimization like AGPSO1, AGPSO2, and AGPSO3 for tuning the Proportional-Integral DVR controller under balanced and nonlinear load conditions. A novel multiobjective function is formulated to express the control performance of the system, which is quantified using three power quality indices such as Total Harmonic Distortion (THD), voltage sag index, and RMS voltage variation. The obtained results are compared with the Proportional-Integral (PI) controller tuned by Ziegler-Nichols (ZN) method and also by Simple Particle Swarm Optimization based PI controlled DVR. The proposed methodology has improved the performance in terms of the considered power quality indices and the simulation has been carried out in MATLAB/Simulink environment.

## 1. Introduction

In recent days, an increased number of sensitive loads have been integrated into the electrical power system. Consequently, both electric utilities and end users are becoming increasingly concerned about the quality and the reliability of electric power. Power quality disturbances have become more serious due to the widespread application of power electronics in the industrial, commercial, and residential sectors. Voltage sag and harmonic distortion are common power quality events that can cause significant destruction to the industrial customers with sensitive loads, resulting in equipment damage, loss of production, and heavy financial loss. Voltage sag is defined as a decrease in the utility supply voltage from 90% to 10% of its nominal value at the power frequency for periods ranging from a half-cycle to a minute. Voltage sag can be symmetrical or unsymmetrical depending on the causes of the sag. Voltage and current waveforms that deviate massively from a sinusoidal form is usually called harmonic distortion. The harmonic distortion of the voltage and the current results in varied problems like greater power losses in distribution systems, malfunctioning of protective devices, electrical, electronic equipment, and problems of electromagnetic interference in communication systems. These disturbances are caused by the use of nonlinear load, energization of large loads, load variations, system faults, and poorly designed systems. According to IEEE 519 standard, THD of voltage should not exceed 5% for distribution systems as keeping low THD values on the system will ensure accurate operation of the equipment and increased life expectancy. There are various conventional and VSC-based compensators available in combating the consequences of voltage disturbances in the distribution systems. Dynamic Voltage Restorer (DVR) is a most cost effective and efficient approach to improve the voltage quality at load side. DVR is a power electronic converter based Distributed-Static Synchronous Series Compensator (DSSSC), designed to protect the sensitive load from supply-side short-term voltage disturbances other than outages. It is connected in series with distribution feeder, at the point of common coupling. By injecting a voltage of desired magnitude and phase angle through a booster transformer, it restores the load-side voltage to be balanced and sinusoidal, even when the source voltage is unbalanced and distorted [1–3]. In [4] four different DVR topologies were analyzed based on methods used to provide sufficient energy during voltage sag condition. Two topologies with no energy storage and two topologies with energy storage are analyzed based on performance, cost, control structure, and complexity. Different types of energy storages that are applied to the DVR system are found in the literature. For instance, Super Magnetic Energy storage systems [5], Super Capacitor [6], Flywheel energy storage [7], Battery Energy Storage Systems [8], Wind energy based compensating system [9] and Photo voltaic based DVR [10] are used to mitigate voltage sags. In this work, battery energy storage system (BESS) is used to provide real power and DVR injects an appropriate voltage to recover the voltage at the sensitive load. Different compensation strategies that have been analyzed for the DVR are the pre-sag compensation [11], the in-phase compensation [11], and the energy-optimized compensation [12]. This paper discusses the application of pre-sag compensation, when designing the DVR compensation system for its effectiveness. This method compensates for exact voltage amplitude and reestablishment of voltage angle of each phase to pre-sag condition.

The inverter is the core component of the DVR, and its control directly affects the performance of the DVR. Over the last few years, major research works have been carried out on control operation of the voltage source inverter in DVR with the objective of obtaining reliable control and fast response procedures to obtain the switch control. Control system based on fast repetitive control of DVR was applied in [13]. Various control approaches such as the PI, fuzzy logic, neural network controller, optimal predictive, sliding mode, and adaptive neurofuzzy inference system are in use [14–21]. Adaptive method based on Hebb learning algorithm for controlling DVR was introduced in [22]. Particle Swarm Optimization based ANFIS for UPQC was used to mitigate voltage sag in [23]. The most common choice for the control of the DVR is the PI controller because of its simple structure and it can offer relatively a satisfactory performance over a wide range of operations. The main problem with this controller is the correct choice of the PI gains, since it is fixed for a particular condition. Further, when it is applied to a complex system such as a power system, the results cannot be acceptable under all operating conditions. To overcome the drawbacks faced in the conventional tuning of controllers and improving the controller performance, a novel method of tuning the PI controllers using Autonomous Group Particle Swarm Optimization algorithms and their variants by considering novel multiobjective function is proposed. The performance of the DVR is evaluated under different operating conditions. In order to validate the better operation of this proposed method, it is compared with the simple Particle Swarm Optimization based PI controller and classical PI controller tuned by Ziegler-Nichols method.

## 2. DVR Modelling

The DVR configuration of the proposed system, mainly, consists of three-phase voltage source inverter, energy storage device, and passive filter and three-phase injection transformer and control circuit to regulate the output voltage of the inverter.

### 2.1. Voltage Source Inverter

Inverter is the main component of DVR. It employs six IGBT power electronic switches with self-commutation by shunt diodes. IGBT combines the fast switching times of the MOSFET with the high voltage capabilities of the GTO. It results in the combination of a medium speed controllable switch capable of supporting the medium power range. Sinusoidal pulse width modulation technique manages the switching operation and through which it can generate a three-phase sinusoidal voltage with any required magnitude, frequency, and phase angle.

### 2.2. Energy Storage Device

Battery energy storage system (BESS) has been considered for inverter side input due to its high operating flexibility and very short response time. These functions provide dynamic power benefits ensuring improved quality of the energy delivered.

### 2.3. Passive Filter

The inverter side filtering is preferred and, using this filtering scheme, the high-order harmonic currents are prevented from penetrating into the series transformer, thus reducing the voltage stress on the transformer.

### 2.4. Series Injection Transformer

Its main function is amplifying the injected voltage and creating an electrical isolation between the voltage source inverter (VSI) and the network. The primary winding of the injection transformer is connected to the inverter side filter while its secondary winding is connected to the distribution network and sensitive load.

### 2.5. Control System

The significant role of the controller is to detect the voltage sag and inject voltage deviation by providing appropriate switching strategies for the inverter. The controller input is an actuating signal which is the difference between the reference voltage and the sensitive load voltage. Switching frequency is in the range of few kHz. Pulse width modulation (PWM) control system is applied for IGBT inverter switching so as to generate a three-phase 50 Hz sinusoidal signals in order to maintain 1 pu voltage at the sensitive load terminal.

## 3. DVR Modes of Operation and Conventional Controller Design

The DVR has two modes of operation, namely, standby mode and boost mode. In standby mode voltage injection is low, so it compensates the voltage sag caused by transformer reactance losses. Most of the time, the DVR will be in this mode. In boost mode, in case of voltage sag caused by any fault conditions or nonlinear load in the distribution system, DVR injects voltage to sensitive load to compensate it.

A controller is required to control or to operate a DVR under boost mode during the fault condition. In [24] the authors reported different types of sag detection methods as applied to DVR system. The primary aim of the control scheme is to maintain constant voltage magnitude at the sensitive load under voltage disturbance condition. In this work, control system uses park transformation to convert the three-phase sensitive load voltage *abc* into *dq* component. During the normal and balanced condition the voltage references are *d*-voltage being unity in pu and *q*-voltage being zero in pu but varies under fault condition. After comparison of *d*-voltage and *q*-voltage with the sensitive load voltage, error signal is generated. Applying this error signal to the PI controller, it is converted into *abc* component through *dq* to *abc* transformation. These *abc* components are given to generate three-phase pulses using pulse width modulation technique. An advantage of the feedback PI controller is that it is so designed as to make the steady state error zero. The general characteristic modelling equation of the PI controller is given by

(1) $$\begin{array}{c} y\left(\;t\;\right)=K_{p}e\left(\;t\;\right)+K_{i}\overset{t}{\underset{0}{\int }}e\left(\;t\;\right)dt, \end{array}$$

where *y*(*t*) is the output of the controller and *e*(*t*) is the error signal. The performance of the PI controller largely depends on the value of *K*_*p*_ and *K*_*i*_. The Ziegler-Nichols method is a highly aggressive method conventionally used more often. Here two PI controllers were used separately for each of the quadrature phases “*d*” and “*q*.” Each of them was separately tuned using Ziegler-Nichols (ZN) method for the identification of the controller setting using the ZN controller setting rules. Using this method the values of *K*_*p*_ and *K*_*i*_ were obtained. For *d* controller they were 40 and 154 and for *q* controller they were 25 and 260, respectively.

## 4. Optimization Problem Formulation

The characterisation of the fitness function of the particle is of paramount importance for the effective performance of an optimization function. The optimization problem is modelled as a minimization problem with three individual indices linearly combined to describe the overall fitness function of a particle.

### 4.1. Minimization of Total Harmonic Distortion (*M*_1_)

The first parameter in the fitness function description is the ability of the current solution in reducing the Total Harmonic Distortion in the system:

(2) $$\begin{array}{c} \min\left(\;M_{1}\;\right)=\min\left\{\;\overset{3}{\underset{j=1}{\sum }}\frac{\sqrt{\sum_{n=2}^{\infty }x_{jn}^{2}}}{x_{jn}}\;\right\}, \end{array}$$

where *x*_*jn*_ is the signal, whose Total Harmonic Distortion is under consideration.

### 4.2. Minimization of Voltage Sag (*M*_2_)

The second parameter under the fitness function description is the sag magnitude of each phase during the fault period. The load terminal containing the voltage sensitive load must operate precisely at 1.0 pu and hence the objective function is designed to minimize the deviation from the operating voltage. If the fault timing ranges from *t*_0_ till *t*_*f*_, then the objective may be mathematically modelled as

(3) min⁡M2=min⁡∑j=131−max⁡xjti,t0≤ti≤tf.

### 4.3. Root Mean Square Voltage Value (*M*_3_)

The third parameter describing the fitness of a particle is the root mean square value of the voltage phasor at the load terminal containing the voltage sensitive load. The objective may be mathematically modelled as shown below if the fault timing ranges from *t*_0_ till *t*_*f*_:

(4) min⁡M3=min⁡∑j=130.707−1tf−t0∫t0tfxjt2,

where *j* = 1,2, 3  and  *x*_*j*_ represents the voltage phasor of all the three phases.

### 4.4. Multiobjective Function

The overall objective function of the optimization problem *M* may be represented as a linear combination of the individual parameters as

(5) $$\begin{array}{c} \min\left(\;M\;\right)=w_{1}M_{1}+w_{2}M_{2}+w_{3}M_{3}, \end{array}$$

where, *w*_1_, *w*_2_, and *w*_3_ are the individual weights allotted to each of the objectives. In this work, the weights allotted to *w*_1_, *w*_2_, and *w*_3_ are 0.4, 0.4, and 0.2, respectively. In addition, the weight factors are assigned based on their importance and may be varied by the power system operator according to the desired performances. Similarly, for the simulation of AGPSO and all its variants, the maximum number of iterations was fixed as 10 with 50 particles per iteration, which is the population size. Owing to various balanced search methodologies, the lesser number of iterations is not an issue for the convergence of the algorithm.

## 5. Particle Swarm Optimization

Swarm intelligence is an innovative distributed intelligent paradigm for solving optimization problems that originally took its inspiration from the biological examples by swarming, flocking, and herd phenomena in vertebrates. The Particle Swarm Optimization (PSO) was proposed by Eberhart and Kennedy in [25]. The idea was inspired by the mechanism of flocking of birds (particles) in search to find the food (solution). Particle Swarm Optimization technique remains as one of the most applied optimization techniques in optimization problems owing to its simplicity in application to multiobjective optimization and its better convergence characteristics. Each particle in the search space moves towards a better solution based on its personal best and the global best achieved till the preceding iterations. The inexpensive computational cost makes this algorithm very popular.

### 5.1. Simple Particle Swarm Optimization (SPSO)

This was the basic method of PSO as proposed by Eberhart and Kennedy in [25]. It uses a number of particles (candidate solutions) which fly around in the search space to find the best solution. The concept of PSO consists of, at each time step, changing the velocity or accelerating each particle towards its pbest (local version of PSO). Acceleration is weighted by a random term, with separate random numbers being generated for acceleration towards pbest locations. PSO is mathematically modelled as follows:

(6) vit+1=wvit+c1×rand×pbesti−xit+c2×rand×gbest−xitxit+1=xit+vit+1,

where *w* is the inertia weight, *v*_*i*_^*t*^ is the velocity of the particle *i* at iteration *t*, *c*_*j*_ is the weighing factor, rand is a random number between 0 and 1, *x*_*i*_^*t*+1  ^ is the current position of particle *i* at iteration *t*, pbest_*i*_ is the personal best position value in the *i*th dimension, and gbest is the best solution so far. The values of the acceleration coefficients *c*_1_ and *c*_2_ are constantly maintained at 2.

### 5.2. Autonomous Group Particle Swarm Optimization (AGPSO)

The Autonomous Group Particle Swarm Optimization was proposed in [26]. Finding the global minimum is a common, challenging task among all the minimization methods. In population-based optimization methods, generally, the desirable way to converge towards the global minimum can be divided into two basic phases. In the early stages of the optimization, the individuals should be encouraged to scatter throughout the entire search space. In the latter stages, the individuals have to exploit information gathered to converge on the global minimum. Considering these points, the autonomous groups' concept is a modification of the conventional PSO. In this method, each group of particles autonomously tries to search the problem space with its own strategy, based on tuning *c*_1_ and *c*_2_.

In conventional PSO, all particles behave in the same way in terms of local and global search, so particles can be considered as a group with one strategy. However, using diverse autonomous groups with a common goal in any population-based optimization algorithm could result in more randomized and directed search simultaneously. Autonomous groups have different strategies to update *c*_1_, so particles could explore the search space locally with different capability than the conventional PSO. Autonomous groups have different strategies to update *c*_2_, so particles could follow social behaviour more autonomously than the conventional PSO. Autonomous groups contain nonlinear patterns such as exponential and logarithmic functions for *c*_1_ and *c*_2_, so they could be more effective. Three different types of methods, namely, AGPSO1, AGPSO2, and AGPSO3, are proposed with each of them having four independent groups searching for the optimal position. A detailed account of all such updating methodologies of individual groups is found in [26].

*Pseudocode for AGPSO*

 Create and initialize a *D*-dimensional PSO
 Divide particles randomly into autonomous groups
 Repeat
 Calculate particles' fitness, Gbest, and Pbest
 For each particle:
 Extract the particle's group
 Use its group strategy to update *c*_1_ and *c*_2_
 Use *c*_1_ and *c*_2_ to update velocities
 Use new velocities to define new positions
 End for
 Until stopping condition is satisfied

## 6. Simulation Results and Discussion

To verify the effectiveness and applicability of the proposed DVR, the distribution system was tested by considering two cases of voltage disturbances and was evaluated using MATLAB/Simulink. The first simulation was carried out in order to see the performance of DVR in compensating 50% balanced voltage sag due to three-phase to ground fault. The second simulation showed the DVR performance by considering nonlinear load. The total period of simulation for each case was 0.12 s. It was assumed that the voltage magnitude of sensitive load was maintained at 1 pu during the voltage sag condition. The system parameters are listed in the Appendix.

The efficient operation of a DVR from the perspective of power quality may be considered based on the performance of the system on certain vital parameters during the occurrence of the fault. The proposed work identifies the following indices regarding the goodness of the system quality.

### 6.1. Voltage Total Harmonic Distortion during Fault

The Total Harmonic Distortion (THD) is an important indication used for the power quality analysis. The fundamental definition of THD is given by

(7) $$\begin{array}{c} {\text{THDV}}_{p}=\frac{\sqrt{\sum_{n=2}^{\infty }V_{pn}^{2}}}{V_{p1}}\cdot 100, \end{array}$$

where *n* is the harmonic order and *p* is the phase order so that *p* = *a*, *b*, *c*. THDV_*p*_ measures the Total Harmonic Distortion with respect to the fundamental frequency. In this proposed method, (7) is used to measure THD with the fundamental frequency of the system being 50 Hz.

The THDV measured can be written as follows:

(8) $$\begin{array}{c} \text{THDV}=\frac{{\text{THDV}}_{a}+{\text{THDV}}_{b}+{\text{THDV}}_{c}}{3}. \end{array}$$

### 6.2. Voltage Sag during Fault

In the event of a fault occurring in one feeder, for reasons such as a short circuit, a high current flows through it along with the supply current. During such a happening, voltage in another feeder will be decreased due to the increased voltage drop across the source reactance. In another event, the nonlinear load connected in one feeder affects the other. During the fault, the voltage magnitude of the corresponding phase drastically drops from the nominal value, and hence operating voltage during the fault period is taken into account.

### 6.3. Detroit Edison Sag Score

This is the first voltage sag index that is used in contracts between utilities and consumers. The Detroit Edison sag score (SS) is defined as follows:

(9) $$\begin{array}{c} \text{SS}=1-\frac{V_{a}+V_{b}+V_{c}}{3}, \end{array}$$

where *V*_*a*_, *V*_*b*_, and *V*_*c*_ are the values of the phase voltages per unit. The larger the sag score, the more severe the disturbances. SS value closer to 0 indicates a good recovered voltage after compensation.

### 6.4. RMS Value during Fault

With the increased disruption of the signal owing to the presence of harmonic content, the RMS value of the signal is also considered as a parameter for analysis of the performance of the DVR. The computation of the RMS value will help in better characterisation of the signal.

**Case 1 (test system with three-phase balanced fault).**

**Figure 1:**
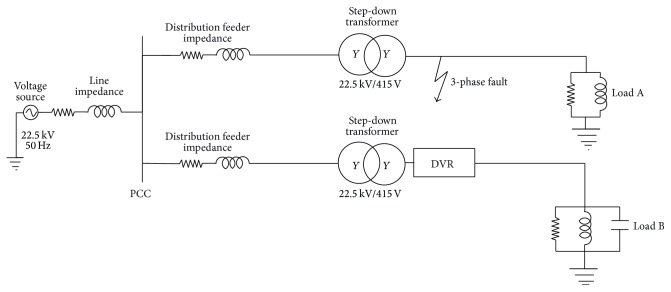
Test system with three-phase balanced fault.

The test system was employed to carry out the simulation of DVR as shown in Figure 1. This system was composed of 22.5 KV, 50 Hz source feeding two distribution lines and two types of loads through two distribution transformers connected in *Y*/*Y* 22.5 KV/415 V. The system included two types of loads: Load A was assumed to be a nonsensitive load while Load B was a sensitive load. The feeder connecting Load B included DVR connected in series. The scenario of simulation in this case was that the balanced three-phase fault was intentionally created near to nonsensitive load using the three-phase fault generator for analyzing the system performance. Voltage sag of nearly 50% was created in all the three phases during the interval of 0.02 s and 0.08 s, assuming the values of fault and ground resistance as 5 Ω and 0.1 Ω, respectively. The performance of the DVR, which in turn is the performance of Load B, was evaluated and the parameters like voltage compensation, Detroit Edison sag score (SS), THDV, and RMS voltage variation index of sensitive load voltage were obtained.

The *K*_*p*_ and *K*_*i*_ parameters of *d* and *q* controllers are tuned using ZN method and various Autonomous Particle Swarm Optimization techniques.  Table 1 compares the performance of the various controllers based on THD improvement. It is obvious that the THDV percentages are consistent with the Total Harmonic Distortion limit of the IEEE Standard 519-1992, for all the controllers. Besides, it was notable that the soft computing based controllers showed better improvement in THDV values in percentage from 15.20% to 22.36% compared with PI-ZN method. After compensation by DVR the sensitive load voltage had a THD of only 0.72% for AGPSO2 based PI controller.

This showed an improved performance of DVR as harmonic compensator. Autonomous Group Particle Swarm Optimization variants showed better improvement in reduction of THD.

**Figure 2:**
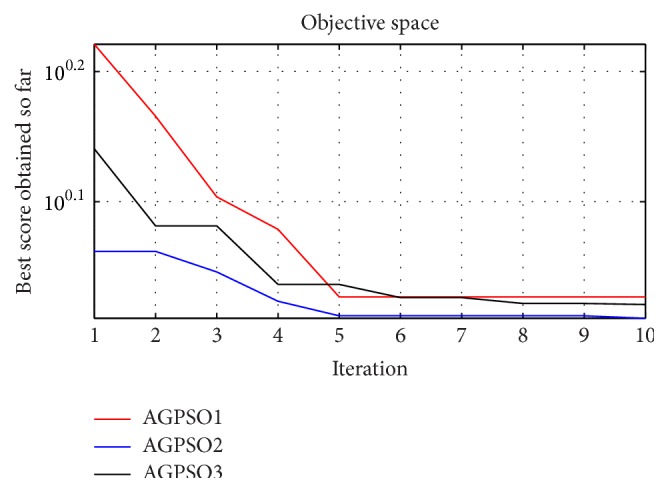
Convergence plot of various AGPSO algorithms.

Table 2 shows the voltage compensation of all the controllers. The sensitive load voltage was regulated at rated value, which showed the satisfactory performance of the DVR for all the optimized controllers. Detroit Edison sag score indicated the closeness of compensation; PSO based PI controller and AGPSO2 based PI controller showed significant improvement in voltage compensation.  Table 3 shows the RMS voltage variation index of all the controllers which also improved and a value of nearly 0.707 volt was achieved. It was observed that AGPSO based PI controller performed better when all aspects were considered. The load voltage was near perfect sinusoidal and the DVR compensated almost 1 pu in all the three phases. The THD of the three phases was reduced compared to all the other controllers. The RMS voltage variation index also improved and a value of nearly 0.707 volt was achieved. From the convergence graph in Figure 2, it is observed that all the AGPSO variants have converged to a nearly similar global fitness value and can also be visualized using their individual performances given in Tables 1–3. However, from Table 4, it may be seen that each of the PSO variants has found a different global best position for the controller setting. Thus the formulated problem is a multiobjective one with multiple optima points.

**Figure 3:**
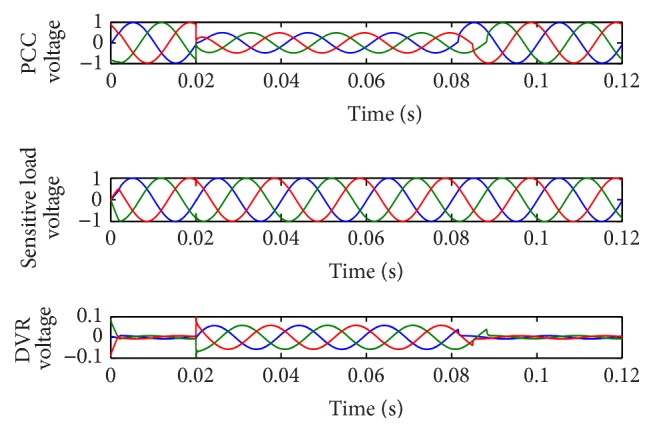
Performance of DVR with 50% three-phase balanced fault using AGPSO2 based PI controller.

Figure 3 shows the PCC voltage, sensitive load voltage, and DVR voltage waveforms using AGPSO2 based PI controller. On observing the waveform, the sensitive load voltage at phase A, phase B, and phase C was maintained at 1.000 pu, 1.000 pu, and 0.9999 pu, respectively, after incorporating the controller with the proposed optimization strategy. The SS value 0.000033 indicates that accurate compensation has been achieved by the controller. The THD of sensitive load voltage after compensation was also reduced at phase A, phase B, and phase C by 0.3265%, 0.8704%, and 0.9913%, respectively. The RMS values of the load voltage at all the three phases were nearly 0.705. The best optimal values obtained by SPSO, AGPSO1, AGPSO2, and AGPSO3 controllers are 1.12, 1.064, 1.0245, and 1.0499, respectively.

**Case 2 (test system with nonlinear load).**

**Figure 4:**
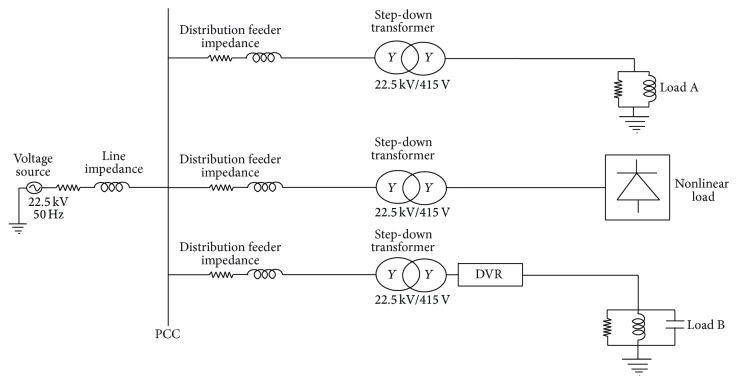
Test system with nonlinear load.

**Figure 5:**
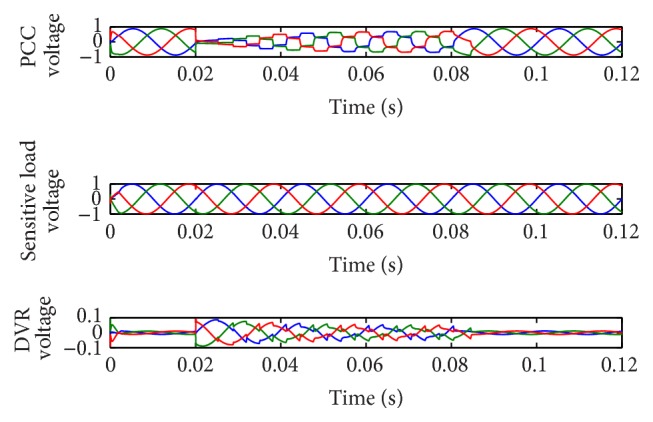
Performance of DVR with nonlinear load using AGPSO1 based PI controller.

In the second case, various loads such as Load A (nonsensitive load), a nonlinear load, and Load B (a sensitive load) were connected at the Point of Common Coupling (PCC), as shown in Figure 4. The nonlinear load is a three-phase diode rectifier bridge with a capacitor bank and resistive load connected in parallel. In this case, DVR performance was evaluated with the same system parameters as considered in Case 1. The total simulation time taken was 0.12 s. Initially, the voltage at PCC was maintained at 1 pu. When the nonlinear load was applied between 0.02 s and 0.08 s, it distorted the voltage and caused unbalanced voltage during the period. Tables 5 and 6 illustrate, respectively, the harmonics and the voltage sag compensation capabilities of DVR considering nonlinear load.  Table 5 shows THDV for PCC voltage of 41.81% and sensitive load voltage of 0.8115% for AGPSO1 based PI controller. This controller showed better performance of about 9.81% improvement with reference to the conventional controller. All the other optimized controllers also performed consistently with slight variations.  Table 6 shows that, in spite of a huge voltage variation at the PCC, DVR is able to provide the sensitive load with necessary voltage for maintaining almost a constant voltage of 1 pu using the controller settings, obtained through various controllers. Tables 5–7 show that all the AGPSO tuning methods outperform the SPSO and ZN tuning methods with respect to the parameters considered. It may be noted that, in most cases, there is almost zero sag and the voltage is maintained perfectly at 1.0 pu. It became evident from results obtained that AGPSO optimized PI controllers performed better for the two main power quality indices considered, the improved voltage compensation capability and reduced THD values. It is observed from Table 7 that the third index, RMS voltage variation, is also improved using the optimized controllers.  Figure 5 shows the PCC voltage, sensitive load voltage, and DVR voltage waveforms using AGPSO1 based PI controller. Best optimal values obtained by SPSO, AGPSO1, AGPSO2, and AGPSO3 were 1.2673, 1.2335, 1.223, and 1.2104, respectively.

The AGPSO optimized PI controllers outperformed their respective counterparts in all aspects.  Table 8 provides the value of *K*_*p*_ and *K*_*i*_ determined by each of the optimization methods. From Table 8, it can be seen that multiple optima exist for the problem and the application of optimization methodology to determine the operating point for each controller is hence justified.

## 7. Conclusion

This study aimed at making improvement in the DVR technology by adopting new control approaches to the system. A comprehensive analysis on the performance of various soft computing based controllers for obtaining better power quality indices was also carried out. From the obtained results, it could be observed that all the proposed artificial intelligence methodologies yielded significant results in comparison with the conventional way of tuning the PI controller using Ziegler-Nichols method. The performance of the conventional controllers and optimized controllers in lieu of system parameters and controller settings was discussed. The application of three versions of Autonomous Group Particle Swarm optimization could substantially improve the performance of the controllers and could achieve the recommended IEEE Standard 512-1992. The inclusion of the controllers in the power circuit was proved to be imperative by the obtained results. The fact that the voltage sag compensation and voltage sag compensation and harmonic mitigation could be achieved by the proposed DVR indicated a significant improvement in the quality of voltage. The work may be extended to study the feasibility of application by optimizing fuzzy and ANFIS controller to the proposed objective functions for the DVR system.

## Figures and Tables

**Table 1:** Harmonic mitigation of three-phase balanced fault.

Tuning method/parameter 1	THD measured at PCC in %				THD measured at sensitive load in %				THD improvement in %
					(compensated)				
	THDV_*a*_	THDV_*b*_	THDV_*c*_	THDV	THDV_*a*_	THDV_*b*_	THDV_*c*_	THDV	
ZN	5.279	5.330	6.507	5.705	0.6629	1.057	1.132	0.9393	—
SPSO	5.254	5.309	6.472	5.678	0.4310	0.9306	1.028	0.7965	15.20
AGPSO1	5.253	5.303	6.465	5.673	0.3660	0.8967	1.015	0.7592	19.17
AGPSO2	5.134	5.390	6.450	5.658	0.3265	0.8704	0.9913	0.7294	22.36
AGPSO3	5.142	5.390	6.457	5.663	0.3376	0.8797	0.9954	0.7375	21.48

**Table 2:** Voltage sag compensation of three-phase balanced fault.

Tuning method/parameter 2	Voltage at PCC in pu				Voltage at sensitive load in pu (compensated)			
	Phase A	Phase B	Phase C	Detroit Edison sag score (SS)	Phase A	Phase B	Phase C	Detroit Edison sag score (SS)
ZN	0.4944	0.4884	0.4884	0.5096	1.007	1.006	1.006	0.006633
SPSO	0.4943	0.4882	0.4883	0.5097	1.001	0.9996	0.9993	0.000033
AGPSO1	0.4946	0.4889	0.4890	0.5091	0.9997	0.9999	1.000	0.000133
AGPSO2	0.4938	0.4876	0.4882	0.5101	1.000	1.000	0.9999	0.000033
AGPSO3	0.4943	0.4881	0.4889	0.5095	0.9996	1.000	0.9999	0.000166

**Table 3:** RMS voltage variation of three-phase balanced fault.

Tuning method/parameter 3	RMS voltage at PCC in volts			RMS voltage at sensitive load in volts		
				(compensated)		
	Phase A	Phase B	Phase C	Phase A	Phase B	Phase C
ZN	0.3448	0.3398	0.3370	0.7046	0.7045	0.7043
SPSO	0.3447	0.3398	0.3370	0.7042	0.7041	0.7042
AGPSO1	0.3448	0.3399	0.3370	0.7048	0.7047	0.7048
AGPSO2	0.3471	0.3406	0.3380	0.7051	0.7047	0.7047
AGPSO3	0.3475	0.3410	0.3383	0.7045	0.7046	0.7048

**Table 4:** Operating points of PI controllers for three-phase balanced fault.

Tuning method	*D* controller		*Q* controller	
	*K*_*p*_	*K*_*i*_	*K*_*p*_	*K*_*i*_
ZN	40	154	25	260
SPSO	32.95	153.77	27.29	140.1
AGPSO1	28.13	200	39.06	130.23
AGPSO2	26.09	200	16.85	0
AGPSO3	26.64	200	23.92	42.51

**Table 5:** Harmonic mitigation of rectifier load.

Tuning method/parameter 1	THD measured at PCC in %				THD measured at sensitive load in %				THD improvement in %
					(compensated)				
	THDV_*a*_	THDV_*b*_	THDV_*c*_	THDV	THDV_*a*_	THDV_*b*_	THDV_*c*_	THDV	
ZN	38.94	45.70	40.80	41.81	0.7076	0.9788	1.013	0.8998	—
SPSO	38.92	45.68	40.79	41.79	0.5972	0.9194	0.9890	0.8352	7.17
AGPSO1	38.93	45.70	40.80	41.81	0.5781	0.8934	0.9630	0.8115	9.81
AGPSO2	38.93	45.69	40.79	41.80	0.5824	0.9069	0.9697	0.8196	8.91
AGPSO3	38.93	45.69	40.79	41.80	0.5786	0.9048	0.9775	0.8203	8.83

**Table 6:** Voltage sag compensation of rectifier load.

Tuning method/parameter 2	Voltage at PCC in pu				Voltage at sensitive load in pu (compensated)			
	Phase A	Phase B	Phase C	Detroit Edison sag score (SS)	Phase A	Phase B	Phase C	Detroit Edison sag score (SS)
ZN	0.6827	0.6947	0.8023	0.2741	0.9984	0.9993	0.999	0.001100
SPSO	0.6835	0.6947	0.8013	0.2735	0.9998	1.000	1.000	0.000066
AGPSO1	0.6833	0.6945	0.8032	0.2730	0.9999	1.000	1.000	0.000033
AGPSO2	0.6834	0.6943	0.8018	0.2735	0.9999	1.000	1.000	0.000033
AGPSO3	0.6833	0.6945	0.8030	0.2730	0.9999	1.000	0.9999	0.000066

**Table 7:** RMS voltage variation of rectifier load.

Tuning method/parameter 3	RMS voltage at PCC in volts			RMS voltage at sensitive load in volts (compensated)		
	Phase A	Phase B	Phase C	Phase A	Phase B	Phase C
ZN	0.3912	0.3820	0.3944	0.7026	0.7024	0.7029
SPSO	0.3914	0.3821	0.3945	0.7030	0.7027	0.7031
AGPSO1	0.3912	0.3820	0.3944	0.7036	0.7033	0.7038
AGPSO2	0.3913	0.3821	0.3945	0.7036	0.7032	0.7037
AGPSO3	0.3913	0.3820	0.3820	0.7036	0.7031	0.7035

**Table 8:** Operating points of PI controllers for rectifier load.

Tuning method	*D* controller		*Q* controller	
	*K*_*p*_	*K*_*i*_	*K*_*p*_	*K*_*i*_
ZN	40	154	25	260
PSO	43.45	162.62	62.23	131.91
AGPSO1	43.11	193.65	37.24	156.41
AGPSO2	41.68	195.69	47.61	219.66
AGPSO3	39.25	200	49.53	78.63

**Table 9:** Summary of design specifications for the test system.

Parameters description	Values with units
Source voltage/frequency	22.5 (kV)/(50 Hz)

Load A parameters	Configuration: *Y* grounded Nominal phase-to-phase voltage *V*_*n*_ (*V*_rms_): 415 V Nominal frequency: 50 Hz Active power: 2500 W Inductive reactive power: 400 (positive var)

Load B parameters	Configuration: *Y* grounded Nominal phase-to-phase voltage *V*_*n*_ (*V*_rms_): 415 V Nominal frequency: 50 Hz Active power: 2500 W Inductive reactive power: 40 (positivevar) Capacitive reactive power: 10 (negative var)

Distribution transformer	Nominal power: 32 (kVA) Winding 1 parameters: 22500 V/star configuration, *R*_1_ = 0.0003 (pu) *L*_1_ = 0.001 (pu) Winding 2 parameters: 415 V/star configuration, *R*_2_ = 0.0003 (pu) *L*_2_ = 0.001 (pu) Magnetization resistance (*R*_*m*_) and magnetization inductance (*L*_*m*_) = 500 (pu)

Series injection transformer	Rated power: 5 kVA/50 Hz Winding 1 parameters: 100 V, *R* = 0.00001 (pu) *X* = 0.0003 (pu) Winding 2 parameters: 1000 V, *R* = 0.00001 (pu) *X* = 0.0003 (pu) Magnetizing branch: *R*_*m*_, *X*_*m*_ = 500 (pu)

PWM generator	Switching frequency = 10 kHz

DC voltage source	200 V

Passive filter	Series filter impedance: *R* = 0.2 Ω, *L* = 6 mH Shunt filter impedance: *R* = 0.2 Ω, *L* = 20 *µ* F

## Appendix

See Table 9.
